# Survey Reveals That Renaming Post-Traumatic Stress ‘Disorder’ to ‘Injury’ Would Reduce Stigma

**DOI:** 10.7759/cureus.38888

**Published:** 2023-05-11

**Authors:** Eugene Lipov

**Affiliations:** 1 Department of Surgery, University of Illinois Chicago, Chicago, USA; 2 Mental Health Clinic, Stella Center, Oak Brook, USA

**Keywords:** stigma, ptsi, post-traumatic stress injury, ptsd, post-traumatic stress disorder

## Abstract

Background

Self-stigmatization has an estimated prevalence of 41.2% among adults with post-traumatic stress disorder (PTSD). Since the name PTSD was introduced, arguments have been made that the term “disorder” may discourage patients from revealing their condition and seeking care. We hypothesize that renaming PTSD to post-traumatic stress injury (PTSI) would reduce the stigma associated with PTSD and improve patients’ likelihood of seeking medical help.

Methods

An anonymous online survey was distributed by the Stella Center (Chicago, IL) between August 2021 and August 2022 to 3000 adult participants, of which 1500 were clinic patients and visitors. Another 1500 invitations were sent out to the Stella Center’s website visitors.

Results

A total of 1025 subjects responded to the survey. The respondents were 50.4% female (51.6% had been diagnosed with PTSD) and 49.6% male (48.4% had been diagnosed with PTSD). Over two-thirds of the respondents agreed that a name change to PTSI would reduce the stigma associated with the term PTSD. Over half of the respondents agreed that it would increase their hope of finding a solution and their likelihood of seeking medical help. The cohort diagnosed with PTSD was most likely to believe in the impact of a name change.

Conclusion

This study provides significant insight into the potential impact of renaming PTSD to PTSI. The biggest effect is likely to be the reduction or elimination of stigma, followed by an increase in the hope of finding successful medical treatment for PTSD. The above changes will likely improve access to care and reduce suicidal ideation in a complex cohort.

## Introduction

Post-traumatic stress disorder (PTSD) is a mental health condition that begins with exposure to actual or threatened death, serious injury, or sexual violence. The presence of intrusion symptoms, such as persistent avoidance of stimuli, negative alterations in cognitions and mood, and marked alterations in arousal and reactivity, are all symptoms that can result from a traumatic event and last for more than a month. The disturbance causes clinically significant distress or impairment in social, occupational, or other important areas of functioning. It is not attributable to the physiological effects of a substance (e.g., medication, alcohol) or another medical condition [1]. The symptoms have behavioral and psychological components and typically affect mood, sleep, and thoughts. The WHO World Mental Health Surveys have estimated the overall international lifetime prevalence of PTSD to be 3.9%, a number which goes up to 5.6% in the subgroup of those exposed to trauma in their lifetime [2]. Nevertheless, PTSD prevalence averaged 10-20% among post-deployment US infantry personnel [3]. In women who had been victims of sexual assault, a 46% lifetime prevalence of PTSD is reported [4]. Though most of the literature focuses on traumatic stressors such as physical and sexual violence, PTSD can also occur in response to global events and natural disasters, to which the geriatric population is especially vulnerable. For instance, nearly 20% of hospitalized COVID-19 patients reported experiencing symptoms of PTSD, with rates as high as 26.9% in elderly COVID-19 survivors [5]. Approximately 25-40% of PTSD patients are expected to remit within a year, although the mean duration of symptoms is upward of 13 years for those with combat-related PTSD [6,7]. PTSD is associated with serious disability, comorbidities, and premature death. The economic costs of PTSD are ample, with work impairment estimated at 3.6 days per month per person with PTSD. One of the major barriers to formal treatment among patients with PTSD is the fear of being stigmatized. In fact, self-stigmatization, which describes the internalization of negative social views and stereotypes, has an estimated prevalence of 41.2% among adults with PTSD [8]. Since the term PTSD was formally introduced in 1980, arguments have been made that the term “disorder” may discourage patients from revealing their condition and seeking care [9-12]. Changing the name to post-traumatic stress injury (PTSI) has been debated since 2011, initially in response to the increasing rates of suicide in the US Military. General Peter Chiarelli determined that service men and women hated the term “disorder” and that it likely prevented them from seeking help [9,11]. In 2012, Drs. Frank Ochberg and Jonathan Shay, two prominent scholars in trauma research, argued to the American Psychiatric Association that the “injury” model is more suitable for describing this condition, as it results from an injury to brain physiology [10-12]. Since then, the proposed name change has received endorsements from veteran and civilian groups, including traumatized populations such as journalists and women who survived rape, incest, and battering [9-12]. Despite these discussions, there is yet no evidence-based consensus on the matter. We hypothesized that renaming PTSD to PTSI would reduce the stigma associated with PTSD and improve patients’ hope of finding a solution.

## Materials and methods

Participants

This study has been exempted from Institutional Review Board (IRB) oversight by Advarra (Protocol number Pro00056144, 08/02/2021). An anonymous online survey was distributed by the Stella Center, between August 2021 and August 2022, to 3000 adult participants, of which 1500 were clinic patients and visitors of the Stella Center. Another 1500 participants interacted with an invitation to the survey made available to the greater public on the Stella Center’s website. The Stella Center is an organization aiming to treat symptoms of emotional trauma and mental health challenges with a global team of medical doctors, psychologists, nurse practitioners, care coordinators, and advocates. There are 29 physical locations throughout the USA. This study was centered around the Chicago, IL site. The Stella Center website receives visitors from all continents, primarily patients diagnosed or suspected of PTSD and their loved ones.

Measures

The survey was administered on Qualtrics. After consenting to participate anonymously in the survey, demographic data on the participants was collected by asking the following questions: What is your sex [Male/Female]; How old are you? [text box]; Which of these statements best describes you? [Active military duty/Military veteran/Other (please describe)]; Have you ever been diagnosed with PTSD? [Yes/No].

Next, the participants were asked four questions regarding whether using the term “PTSI” rather than “PTSD” would reduce stigma and improve subjects’ hope and likelihood of seeking treatment. This section of the survey constituted of the following statements: The name PTSI would reduce the stigma associated with the term PTSD; The name PTSI would increase my hope in finding a solution for my symptoms; The name PTSI would increase my likelihood of seeking medical help; The name PTSI would increase my likelihood of seeking interventional treatments such as transcranial magnetic stimulation (TMS) or stellate ganglion block (SGB). The subjects’ strength of agreement with the statements was assessed using a five-point Likert scale (strongly disagree/ somewhat disagree/ neither agree nor disagree/ somewhat agree/ strongly agree).

Data analysis

Data analysis was performed using SPSS (IBM). Two-tailed t-tests were used to statistically analyze the demographic variables of the subjects. Two-sided Pearson Chi-square tests were performed to statistically analyze survey responses by sex or PTSD diagnosis. A p-value of less than 0.05 was considered statistically significant.

## Results

Of the 3000 survey invitations distributed, a total of 1353 responses were received. One thousand twenty-five of these survey responses were complete, with answers to every question, and were used in this analysis. The average age of the female respondents (50.4%) was 37.5 ±13.3, and the average age of the male respondents (49.6%) was 46.2 ±12.6 (p<0.001). A total of 11.9% of female respondents had a military status (5.1% were active military and 6.8% were veterans), and 62.7% of male respondents had a military status (13.9% were active military and 48.8% were veterans) (p<0.001). Furthermore, 51.6% of the female subjects had been diagnosed with PTSD, along with 48.4% of the male subjects (p=0.445). Of note, age and military status were significantly different between the survey responders who had been diagnosed with PTSD and those who had not been diagnosed (p<0.001) (Table 1).

**Table 1:** Demographic distribution by PTSD diagnosis. PTSD: Post-traumatic stress disorder.

		Diagnosed with PTSD	Not diagnosed with PTSD	P-value
Sex	Male	236 (48.4%)	267 (50.8%)	0.445
	Female	252 (51.6%)	259 (49.2%)	
Age	<30	108 (21.0%)	159 (29.3%)	<0.001
	31-40	127 (24.7%)	123 (22.7%)	
	41-50	145 (28.2%)	96 (17.7%)	
	51-60	95 (18.4%)	103 (19.0%)	
	>61	40 (7.8%)	62 (11.4%)	
Military status	Active military duty	29 (5.8%)	73 (14.0%)	<0.001
	Military veteran	188 (37.6%)	114 (21.8%)	
	Other	283 (56.6%)	336 (64.2%)	

**Figure 1:**
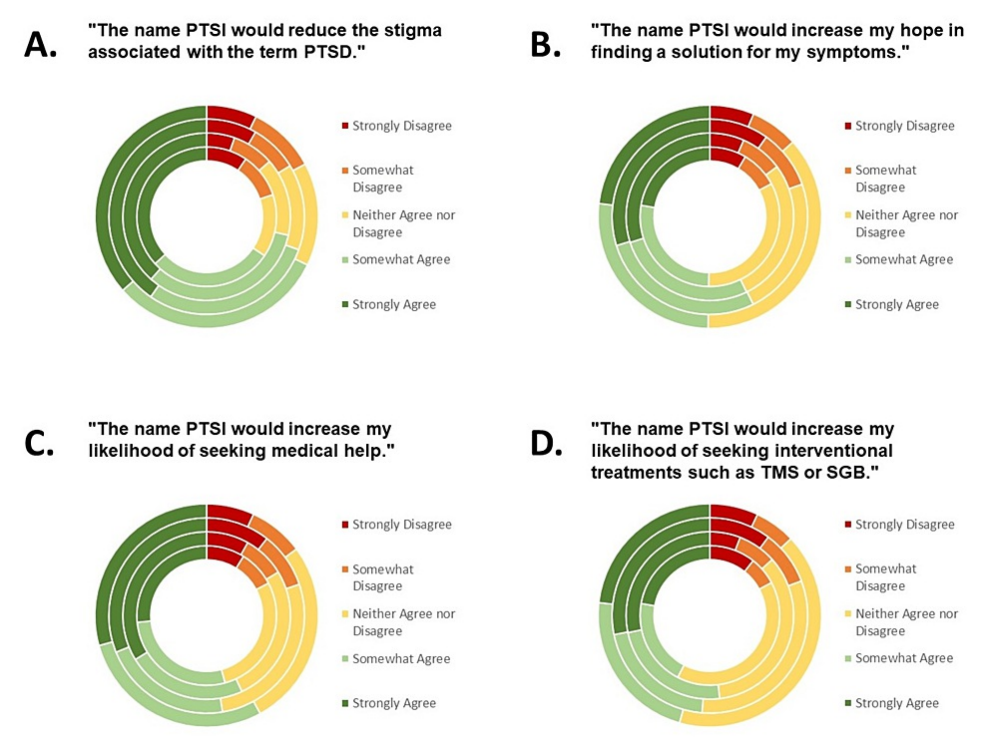
Strength of agreement of survey respondents with statements regarding renaming PTSD to PTSI. Responses are illustrated by groups from inner to outer circles: males, females, subjects with a PTSD diagnosis, and subjects without a PTSD diagnosis. Respondents answered on a Likert scale to assess the strength of agreement with the following statements. A. "The name PTSI would reduce the stigma associated with the term PTSD." B. "The name PTSI would increase my hope in finding a solution for my symptoms." C. "The name PTSI would increase my likelihood of seeking medical help." D. "The name PTSI would increase my likelihood of seeking interventional treatments such as transcranial magnetic stimulation (TMS) or stellate ganglion block (SGB)." PTSD: Post-traumatic stress disorder;  PTSI: Post-traumatic stress injury.

Approximately 69% of survey respondents agreed with the statement, "The name PTSI would reduce the stigma associated with the term PTSD (Figure 1A)." The respondents who had been diagnosed with PTSD reported the highest rates of strong agreement (40%). Among these respondents, rates of agreement were influenced by sex (p=0.024), military status (p=0.02), and age (p=0.013) (Table 2). Only 15% of respondents were ambivalent on the topic.

**Table 2:** Strength of agreement with statements regarding renaming PTSD to PTSI among survey respondents diagnosed with PTSD. PTSD: Post-traumatic stress disorder;  PTSI: Post-traumatic stress injury; TMS: Transcranial magnetic stimulation; SGB: Stellate ganglion block.

		Sex			Military Status				Age					
		Male	Female	P-value	Active	Veteran	Other	P-value	<30	31-40	41-50	51-60	>61	P-value
“The name PTSI would reduce the stigma associated with the term PTSD.”	Strongly Disagree	10%	6%	0.024	10%	12%	6%	0.020	5%	6%	12%	7%	15%	0.013
	Somewhat Disagree	11%	7%		7%	12%	6%		11%	11%	8%	1%	7%	
	Neither Agree nor Disagree	12%	16%		7%	14%	14%		14%	13%	12%	17%	15%	
	Somewhat Agree	25%	34%		41%	24%	31%		41%	31%	26%	24%	20%	
	Strongly Agree	43%	37%		34%	38%	44%		30%	39%	43%	51%	44%	
“The name PTSI would increase my hope in finding a solution for my symptoms.”	Strongly Disagree	11%	8%	0.454	14%	12%	8%	0.067	8%	9%	12%	5%	12%	0.467
	Somewhat Disagree	9%	9%		3%	11%	10%		13%	12%	8%	5%	10%	
	Neither Agree nor Disagree	26%	22%		21%	28%	20%		19%	24%	20%	29%	27%	
	Somewhat Agree	26%	30%		41%	25%	27%		33%	22%	29%	29%	27%	
	Strongly Agree	28%	31%		21%	24%	35%		26%	34%	32%	31%	24%	
“The name PTSI would increase my likelihood of seeking medical help.”	Strongly Disagree	13%	8%	0.502	10%	15%	7%	0.093	9%	12%	12%	5%	13%	0.810
	Somewhat Disagree	8%	9%		7%	8%	10%		12%	10%	8%	4%	8%	
	Neither Agree nor Disagree	29%	27%		38%	29%	26%		26%	29%	24%	31%	33%	
	Somewhat Agree	21%	22%		17%	23%	21%		21%	20%	23%	26%	18%	
	Strongly Agree	29%	34%		28%	25%	36%		31%	29%	33%	33%	30%	
“The name PTSI would increase my likelihood of seeking interventional treatments such as TMS or SGB.”	Strongly Disagree	14%	7%	0.042	14%	15%	6%	0.024	9%	12%	11%	5%	12%	0.210
	Somewhat Disagree	8%	10%		7%	8%	10%		11%	6%	10%	5%	12%	
	Neither Agree nor Disagree	34%	31%		34%	36%	29%		30%	35%	24%	39%	44%	
	Somewhat Agree	17%	24%		14%	18%	23%		26%	20%	20%	21%	12%	
	Strongly Agree	27%	29%		31%	23%	31%		24%	27%	34%	30%	20%	

When asked whether: "The name PTSI would increase my hope in finding a solution for my symptoms," 53% of subjects agreed (Figure 1B). Respondents who had been diagnosed with PTSD reported the highest rates of agreement (57%) that renaming PTSD would increase their hope. PTSD diagnosis may be connected to feelings of hope; a higher percentage of responders with a PTSD diagnosis strongly agreed with this (30% vs. 23%), whereas a higher percentage of responders without a PTSD diagnosis neither agreed nor disagreed (37% vs. 23%) (p<0.001). Next, subjects were asked whether "The name PTSI would increase my likelihood of seeking medical help." Once again, more than half of the respondents agreed with the statement (55%) (Figure 1C). When asked more specifically whether "The name PTSI would increase my likelihood of seeking interventional treatments such as transcranial magnetic stimulation (TMS) or stellate ganglion block (SGB)," the rates of agreement reached 47%, counting 49% of responders with a PTSD diagnosis (Figure 1D). Among the respondents with a PTSD diagnosis, rates of agreement with this survey question were influenced by sex (p=0.042) and military status (p=0.024) (Table 2).

## Discussion

This is the first study of its kind, demonstrating that renaming PTSD to PTSI is likely to have a significant impact by reducing the stigma associated with PTSD, improving patients' hope in finding a treatment and increasing the patient population seeking medical help. The results of this survey are striking, with over two-thirds of the 1025 respondents agreeing that a name change to PTSI would reduce the stigma associated with the term PTSD, and only 15% of respondents feeling undecided on the topic. With nearly half of the surveyed population having a PTSD diagnosis, it is evident that the stigma associated with the term "disorder" is recognized by those with and without a diagnosis. The respondents with a PTSD diagnosis were most likely to strongly agree that the name change would decrease stigma, particularly among men and those in the age group 51-60 years. The agreement was slightly less pronounced among veterans and those aged >61 years. Furthermore, the respondents with a PTSD diagnosis were most likely to strongly agree that the name change would increase their hope of finding a solution. However, again, this was less pronounced among veterans. PTSD is known to be associated with hopelessness, as well as suicidal ideation, in response to feelings of guilt [13,14]. As our results suggest that a name change may result in decreased stigma, we further hypothesize that it may also reduce the rates of suicidal ideation among patients with PTSD. Indeed, suicide prevention may be achieved by reducing perceived stigma and self-stigma [9,15]. Self-stigmatization occurs when an individual agrees with a public stereotype of people with mental illness and applies it to themselves [16]. Among people with PTSD, self-stigma has an estimated prevalence of 41.2% [8]. The same cohort found a 68.5% prevalence of alienation, a 12.4% prevalence of stereotype endorsement, a 53.6% prevalence of discrimination experience, a 60.3% prevalence of social withdrawal, and a 28.9% prevalence of stigma resistance. No association was found between self-stigma and gender, age, sexual trauma, or military trauma. However, self-stigma was associated with lower income and higher levels of anxiety, depression, and traumatic stress symptoms. High levels of self-stigma are typically associated with low self-esteem and quality of life and may interfere with rehabilitation goals [17]. A study found that treatment-seeking veterans with combat-related PTSD believe that they are stigmatized by the public, most commonly with stereotypes such as "dangerous" or "violent" [17]. This group of veterans was also found to believe that the public would hold them responsible for causing their own illness because they volunteered for military duty. In fact, military stigma, specifically, has been defined as "a set of beliefs based on a service member's military and prior civilian enculturation that seeking mental health treatment would be discrediting or embarrassing, cause harm to military career prospects, or cause peers or superiors to have decreased confidence in the service member's ability to perform assigned duties" [18].

Stigma has been repeatedly highlighted as the key barrier to help-seeking behaviors [19]. Studies on mental health disorders have shown that self-stigmatization may decrease treatment-seeking and undermine adherence to treatment recommendations [17]. In fact, most veterans choose to cope without treatment, relying on their own resilience and, in some cases, on substance use. As a result of refraining from seeking mental health services due to stigma, patients with PTSD may endure extreme and life-threatening consequences such as depression, substance abuse, and suicide [20,21]. Beyond self-stigma, perceived stigma represents negative beliefs that society as a whole may hold against mental illness. Studies on the efficacy of interventions for reducing such stigma have mainly shown small-to-medium immediate effects and call for more research to determine how to sustain these effects in the long term [22, 23]. A review including 62 randomized controlled trials identified the most effective interventions for reducing stigma regarding severe mental illness to be contact interventions (exposure to individuals with severe mental illness to reduce anxiety and increase empathy) and educational interventions (correcting inaccurate stereotypes with factual information) [22]. Another large review, including 80 studies and eight systematic reviews on mental-health-related stigma and discrimination, obtained similar results, finding that social contact was the most effective type of intervention to improve stigma-related attitudes and knowledge [23]. Once again, however, the evidence for the long-term benefit of these interventions is weak. Until 2013, there was little evidence suggesting the superiority of either pharmacotherapy or psychotherapy in treating PTSD [24]. There is now strong evidence backing non-pharmacological approaches such as manualized trauma-focused psychotherapy and cognitive processing therapy. As for pharmacological approaches, there is strong evidence for the use of fluoxetine, paroxetine, sertraline, and venlafaxine. Stigma remains a major barrier to treatment-seeking behaviors. When it comes to discussing possible medical treatments, our study found that 55% of respondents agreed that the name change would increase their likelihood of seeking medical help. Among the respondents with a PTSD diagnosis, this finding was consistent regardless of sex, age, and military status. Our study also found that 47% of respondents agreed that the name change would increase their likelihood of seeking interventional treatments in particular. Among the respondents with a PTSD diagnosis, this agreement was less pronounced among female responders and veterans. Randomized clinical trials have shown the safety and efficacy of interventional treatments such as TMS and SGB in treating PTSD symptoms [25-27]. Although the quality of evidence remains low, a need for large, well-designed clinical trials is clear. The use of an anonymous survey certainly has advantages in the ease of collecting data from a large number of respondents. Nonetheless, some limitations are also attributed to this study type, as surveys make communicating and capturing an emotional response harder. On this note, the positive phrasing of the statements assessed using a Likert scale may have led the participants to respond in a certain way. Additionally, there is a possibility of selection bias, as our survey was distributed to a limited population, and some demographic characteristics were significantly different between groups. Another limitation is that we were not able to verify the presence of a formal PTSD diagnosis, as it was self-reported by the survey responders. Due to the brevity of the survey, little demographic data was collected from the participants, such as socioeconomic status, education level, race, and co-morbidities. Our sampled population may not reflect the general population.

## Conclusions

This study provides some significant insight and evidence into a potential reversal of the stigma associated with PTSD as a "disorder." These results indicate that renaming the condition to PTSI would decrease the stigma associated with this condition. Increasing the hope of medical treatment and improving the care for this patient population may lead to higher patient acceptance of current and new treatments such as SGB and TMS, hopefully decreasing misery due to PTSD and the occurrence of suicidal behavior in this complex cohort.
